# Association Between Marital Satisfaction and Physical Activity Among Married Saudi Adults Aged 25–59 Years Attending Primary Health Care Centers in Taif, Saudi Arabia: A Cross-Sectional Study

**DOI:** 10.7759/cureus.116601

**Published:** 2026-09-20

**Authors:** Meteb A Alghamdi, Nesrin K Abd El-Fatah, Yousef Alsulaimani

**Affiliations:** 1 Preventive Medicine, King Abdulaziz Hospital, Taif, SAU; 2 Public Health, High Institute of Public Health - Alexandria University, Alexandria, EGY; 3 Preventive Medicine, Taif Health Cluster, Taif, SAU

**Keywords:** marital satisfaction, married adults, physical activity, relationship quality, saudi arabia.

## Abstract

Background: Marital satisfaction is a key indicator of psychological well-being and family stability. Emerging evidence suggests that lifestyle behaviors, particularly physical activity, may be related to relationship quality, but data from Middle Eastern populations remain limited. This study examined the association between marital satisfaction and physical activity among married Saudi adults aged 25-59 years attending primary health care centers in Taif.

Methods: A cross-sectional study was conducted among 494 married Saudi adults aged 25-59 years who had been married for at least two years and were attending primary health care centers in Taif, Saudi Arabia. Participants were selected using a multistage stratified random sampling technique. Marital satisfaction was measured with the Relationship Assessment Scale (RAS) and physical activity with the International Physical Activity Questionnaire-Short Form (IPAQ-SF). Associations were examined using the Pearson chi-square test or the Fisher-Freeman-Halton exact test (for tables with sparse cells), with the bias-corrected Cramér’s V as an effect size, and using binary logistic regression (penalized for sparse data) to estimate crude and adjusted odds ratios (OR and aOR) with 95% confidence intervals (CIs) for high marital satisfaction.

Results: High marital satisfaction was reported by 363 (73.5%) participants, and 367 (74.3%) were in the low overall IPAQ-SF physical activity category. Marital satisfaction was significantly associated with vigorous activity (χ² = 53.41, p < 0.001, V = 0.22), moderate activity (χ² = 21.73, p = 0.002, V = 0.13), walking (χ² = 30.71, p < 0.001, V = 0.16), and the overall IPAQ-SF category (χ² = 21.43, p < 0.001, V = 0.13); all effect sizes were small. In adjusted analyses, a higher overall IPAQ-SF category was associated with lower odds of high marital satisfaction (aOR per category 0.66, 95% CI 0.44-1.00, p = 0.048), and more frequent vigorous activity was inversely associated with high marital satisfaction (aOR per level 0.44, 95% CI 0.24-0.79, p = 0.006). Moderate activity (aOR 1.12, 95% CI 0.77-1.62) and walking (aOR 1.24, 95% CI 0.87-1.76) were not independently associated with marital satisfaction. Results were unchanged after excluding two participants reporting more than one wife.

Conclusions: In this cross-sectional sample, more frequent vigorous activity and membership in the very small high overall IPAQ-SF activity category were inversely associated with high marital satisfaction, whereas moderate activity and walking showed no independent association; all associations were modest. Because of the cross-sectional design, the temporal direction cannot be determined, and the findings represent associations rather than causal effects.

## Introduction

Marital satisfaction is a key component of family stability and psychological well-being [1], reflecting an individual’s subjective evaluation of the quality and functioning of their marital relationship [1, 2]. It is a multidimensional construct affected by emotional, interpersonal, social, and economic factors and has been widely studied within psychology, public health, and social sciences [3]. High marital satisfaction has been consistently associated with better mental health outcomes, improved quality of life, and stronger family cohesion, whereas low satisfaction is linked to psychological distress, conflict, and increased risk of marital dissolution [4]. The determinants of marital satisfaction are complex and culturally sensitive, varying across populations and socioeconomic contexts. Previous research has identified multiple contributing factors, including demographic characteristics, duration of marriage, educational level, income, interpersonal communication, and health-related conditions [5]. In addition, lifestyle behaviors have recently gained attention as potential modifiable correlates of marital quality - particularly physical activity, which is increasingly recognized for its broad psychological and relational benefits [6].

Physical activity, defined as any bodily movement produced by skeletal muscles that requires energy expenditure, is a well-established determinant of physical and mental health [7]. According to the World Health Organization, regular moderate-to-vigorous physical activity reduces the risk of chronic diseases and is also associated with improved mental health outcomes, including reduced symptoms of depression and anxiety and enhanced emotional well-being [8]. These psychological benefits may indirectly contribute to better relationship functioning through improved mood regulation, stress reduction, and increased energy levels. The International Physical Activity Questionnaire (IPAQ) is one of the most widely used instruments for assessing physical activity across populations and has demonstrated acceptable validity and reliability in diverse settings [9].

Despite growing evidence linking physical activity to mental health, less attention has been given to its potential association with marital satisfaction, particularly in Middle Eastern populations. Marital satisfaction has been measured using several standardized tools, among which the Relationship Assessment Scale (RAS) is a brief and validated instrument designed to assess global relationship quality in intimate partnerships [10]. It captures overall satisfaction, perceived relationship quality, and emotional evaluation of the marital bond. Although previous studies in different cultural settings have suggested that physically active individuals tend to report better psychological well-being and interpersonal functioning, evidence directly examining the relationship between physical activity and marital satisfaction remains limited and inconsistent [11]. Furthermore, little is known about this association among married adults in Saudi Arabia, where cultural, social, and lifestyle factors may uniquely shape both marital dynamics and health behaviors. Therefore, this study aimed to investigate the association between marital satisfaction and physical activity among married Saudi adults attending primary health care centers in Taif City, Saudi Arabia. We hypothesized that physical activity would be associated with marital satisfaction in this population; because the study was cross-sectional, no direction of effect was assumed.

## Materials and methods

Study Design and Setting

A cross-sectional study design was used to examine the association between marital satisfaction and physical activity among married Saudi adults attending primary health care (PHC) centers in Taif City, Saudi Arabia.

Study Participants

The target population included married men and women aged 25-59 years who had been married for at least two years.

Eligibility Criteria

Participants who were non-Saudi, outside the specified age range, married for less than two years, infertile, menopausal, or who had physical disabilities were excluded to reduce confounding factors that could influence marital satisfaction or physical activity levels.

Sampling and Sample Size

The sample size was calculated using Epi Info™ version 7 as a precision-based estimate for a single proportion. In the absence of previous data on marital satisfaction among married adults in the Taif region, an expected proportion of 50% was assumed to maximize the required sample. Using a 95% confidence level and a 5% margin of error, the minimum required sample was 384 participants. Because this was a precision-based calculation for estimating a proportion rather than a power-based hypothesis-testing calculation, statistical power (and a corresponding beta error) was not an input to the calculation. After increasing the number to allow for incomplete responses, the final target sample was 500 participants.

A multistage stratified random sampling technique was applied. Taif City was first divided into five geographic sectors (north, south, east, west, and central); primary health care centers were then randomly selected within each sector (eight centers in total), and both male and female participants were randomly recruited from outpatient clinics, follow-up visits, and well-baby clinics.

Data Collection

The questionnaire was administered electronically using Google Forms. A QR code linked to the questionnaire was provided in person to eligible visitors attending the selected primary health care centers, allowing participants to access and complete the questionnaire using their own mobile devices. For participants who preferred receiving a direct link, the questionnaire link was sent via WhatsApp. Data were collected over a nine-month period. The questionnaire comprised 40 items in four sections: the first two sections included 24 items on sociodemographic, household, health-related, marital, and psychosocial characteristics (including the two-item Patient Health Questionnaire-2 [PHQ-2] and single-item self-rated sleep-quality and health ratings); the third section included 9 marital satisfaction items; and the fourth section included 7 IPAQ-SF physical activity items. One item (number of wives) was shown only to male participants. The estimated completion time was approximately 10-15 minutes, with most of the time required for the final section; individual completion times were not recorded.

The first section covered sociodemographic, household, and behavioral characteristics (age, sex, educational level, household size, monthly income, and tobacco use), with items adapted from the World Health Organization STEPwise approach to noncommunicable disease risk-factor surveillance (WHO STEPS) instrument [12]. The second section assessed health-related, marital, and psychosocial characteristics, including family history of disease, chronic illness, marriage duration, exposure to abuse during the previous 12 months, and negative life circumstances affecting the relationship, together with single-item self-rated sleep quality and overall health and depressive symptoms measured using the PHQ-2 [13]. The third section comprised the RAS, and the fourth the IPAQ-SF. Before the main data collection, a pilot study was conducted on 20 participants to assess the clarity, feasibility, and appropriateness of the questionnaire; minor adjustments were made based on feedback, and pilot data were not included in the final analysis.

Study Instruments and Measures

Marital satisfaction was assessed using the Relationship Assessment Scale (RAS) developed by Susan S. Hendrick. The RAS is a brief, self-administered instrument designed to measure global satisfaction within intimate relationships. Nine relationship items were administered; the seven items that constitute the standard RAS were used to compute the total score, and the two additional items (satisfaction with closeness and harmony, and how often the partner meets one’s needs) were collected but not included in the RAS total. Each item is rated on a 5-point scale. Two of the seven scored items (items 4 and 7) are negatively worded and were reverse-scored before summation. The seven items were summed to obtain a total RAS score ranging from 7 to 35, with higher scores indicating greater relationship satisfaction. Participants were categorized as having low (7-14), average (15-21), or high (22-35) marital satisfaction [10,14]. In this sample, the observed total RAS score ranged from 10 to 35 (mean 24.6, SD 6.8), and internal consistency was high (Cronbach’s alpha = 0.94). For the primary analysis, marital satisfaction was dichotomized into high versus low/average satisfaction.

Physical activity was assessed using the Arabic short version of the International Physical Activity Questionnaire (IPAQ-SF), which was translated for this study (see Translation of the Questionnaires). The IPAQ-SF assesses vigorous-intensity activity, moderate-intensity activity, walking, and sitting time during the previous seven days. The IPAQ-SF is usually scored in metabolic equivalent (MET)-minutes per week, which requires both the number of days and the number of minutes spent in each activity. In this study, most participants reported the number of days of activity but did not report the duration in minutes, so MET-minutes per week could not be calculated, and the standard MET-based classification could not be applied. Physical activity was therefore classified from the reported weekly frequency of activity. For each activity type, participants were grouped by the number of days per week, and an overall IPAQ-SF physical activity category (low, moderate, or high) was derived by combining these frequencies, with higher-intensity activities contributing more to the overall category. This variable is referred to as the overall IPAQ-SF physical activity category (in brief, the overall IPAQ-SF category) throughout this article. Sedentary behavior was represented by daily sitting time, which participants reported as free text; these responses were converted to hours per day for descriptive summary, and “do not know” answers (n = 7) were treated as missing. Sitting time was analyzed separately and was not included in the physical activity classification [15,16].

All measurement instruments used in this study are publicly available and free to use for non-commercial academic and research purposes, and each was cited to its original source; accordingly, no separate copyright permission was required. The WHO STEPS instrument [12] is a publicly available tool that may be used for non-commercial research with attribution; the PHQ-2 [13] is in the public domain; the IPAQ-SF [15,16] is distributed under a Creative Commons Attribution license and may be used and translated without prior permission; and the RAS [10,14] is freely available for non-commercial research use with citation to its original publications, and its full item set was not reproduced here.

Translation of the Questionnaires

All questionnaires were originally written in English and were translated into Arabic using a forward and backward translation process. Five bilingual individuals first translated the English questionnaires into Arabic, and their translations were reviewed and combined into one Arabic version. This version was then back-translated into English and compared with the original to confirm that the meaning of each item was maintained; discrepancies were discussed and resolved. Finally, five people fluent in both Arabic and English reviewed the final Arabic version for clarity and consistency, and minor changes were made before use.

Statistical Analysis

Data were analyzed using IBM SPSS Statistics version 28 (IBM Corp., Armonk, NY, USA) and Python (statsmodels and SciPy). Categorical variables were summarized as frequencies and percentages, and continuous variables as mean and standard deviation (SD). Associations between categorical variables and marital satisfaction were examined using the Pearson chi-square test. When more than 20% of cells had expected counts below five, the Fisher-Freeman-Halton exact test was used instead; its p-value, based on ordering the tables by their exact probability under the null hypothesis, was obtained by Monte Carlo estimation (1,000,000 tables sampled with the observed row and column totals held fixed; random seed 2024) in Python (NumPy and SciPy). For these tables, the Pearson χ² statistic and its degrees of freedom are reported for descriptive purposes only, while the accompanying p-value is the Monte Carlo estimate of the exact p-value. For each association, the bias-corrected Cramér’s V was reported as an effect-size measure, and significance was interpreted together with the effect size rather than by the p-value alone. Values below 0.001 are reported as p < 0.001.

Binary logistic regression was used to estimate associations with high marital satisfaction (high versus low/average). The primary exposure was the overall IPAQ-SF physical activity category (low as the reference), and the three activity domains (vigorous, moderate, and walking) were examined in separate secondary models. Because the high physical activity category and the higher-frequency activity categories were small, several models showed sparse-data instability and quasi-complete separation; Firth’s penalized likelihood was therefore used for the categorical models, and, where the reference category was very small, the frequency of each domain was additionally modeled as an ordinal (per-category) term to provide a stable summary estimate. Crude and adjusted odds ratios (OR and aOR) with 95% CIs are reported. Confounders were selected a priori on the basis of subject-matter knowledge and their plausible relationship with both physical activity and marital satisfaction (age, sex, monthly income, and chronic health problems), rather than by univariate significance or stepwise selection; body mass index and smoking were not included in the primary model because they were considered potential consequences of, or downstream from, physical activity rather than confounders of the exposure-outcome relationship. Effect modification by sex was assessed by adding a sex × physical activity interaction term to the same penalized (Firth) logistic model, with physical activity entered as the ordinal per-category term; the interaction was evaluated using the Wald test for this term. Two sensitivity analyses were performed: one excluding the two participants who reported more than one wife (N = 492), and one modeling marital satisfaction as an ordinal outcome (low < average < high). Analyses of physical activity by sex, age group, and body mass index category; of smoking by sex; and of abuse in relation to marital satisfaction were pre-specified to address specific questions and are reported descriptively with the appropriate test and effect size. These subgroup, interaction, and sensitivity analyses were secondary and are interpreted cautiously.

## Results

Of the 500 respondents who completed the questionnaire, six who reported a marriage duration of less than one year did not meet the eligibility criterion of being married for at least two years and were excluded, leaving 494 participants in the analytic sample. The sociodemographic characteristics (Table 1), and the health-related, marital, and household characteristics (Table 2). Most participants were aged 31-40 years (424, 85.8%), and 305 (61.7%) were men. University education was reported by 473 (95.7%) participants, and 396 (80.2%) reported a monthly family income of 10,000-15,000 Saudi Riyals. Most participants lived in urban areas (379, 76.7%), were non-smokers (411, 83.2%), and were overweight (388, 78.5%). A chronic health problem was reported by 367 (74.3%) participants. Relationship-related experiences, psychosocial factors, and self-rated sleep and health (Table 3).

**Table 1:** Sociodemographic characteristics of the study participants (N = 494). Values are n (%). SR: Saudi Riyals.

Characteristic	Category	No. (%)
Age (years)	25–30	30 (6.1)
	31–40	424 (85.8)
	41–50	38 (7.7)
	51–59	2 (0.4)
Partner’s age (years)	25–30	57 (11.5)
	31–40	428 (86.6)
	41–50	9 (1.8)
Sex	Male	305 (61.7)
	Female	189 (38.3)
Educational level	Diploma	17 (3.4)
	University	473 (95.7)
	Post-graduate	4 (0.8)
Partner’s education	Secondary or below	18 (3.6)
	Diploma	61 (12.3)
	University	412 (83.4)
	Post-graduate	3 (0.6)
Consanguineous marriage	Yes	173 (35.0)
	No	321 (65.0)
Household size	2–3	61 (12.3)
	4–6	425 (86.0)
	7–8	8 (1.6)
Monthly family income (SR)	3000–6000	9 (1.8)
	6000–10000	70 (14.2)
	10000–15000	396 (80.2)
	15000–20000	13 (2.6)
	>20000	6 (1.2)
Residence	Urban	379 (76.7)
	Rural	115 (23.3)

**Table 2:** Health-related, marital, and household characteristics of the study participants (N = 494). Values are n (%). † Family history of disease allowed more than one response; therefore, the categories may add up to more than 100%.

Characteristic	Category	No. (%)
Smoking status	Non-smoker	411 (83.2)
	Ex-smoker	20 (4.0)
	Current smoker	63 (12.8)
Family history of disease †	None	127 (25.7)
	Hypertension	150 (30.4)
	Diabetes mellitus	134 (27.1)
	Cardiac disease	35 (7.1)
	Tumor	29 (5.9)
	Renal disease	27 (5.5)
	Physical disability	10 (2.0)
	Hematological disease	3 (0.6)
Chronic health problem	No	127 (25.7)
	Yes	367 (74.3)
Number of wives (men, n = 305)	1 wife	303 (99.3)
	2 wives	2 (0.7)
Marriage duration (years)	1–3	35 (7.1)
	4–5	83 (16.8)
	6–7	173 (35.0)
	8–10	175 (35.4)
	>10	28 (5.7)
Housing	Owner-occupied	286 (57.9)
	Rented	202 (40.9)
	With family	6 (1.2)
Body mass index	Normal weight	98 (19.8)
	Overweight	388 (78.5)
	Obese	8 (1.6)

**Table 3:** Relationship-related experiences, psychosocial factors, sleep quality, and self-rated health of the study participants (N = 494). Values are n (%) unless otherwise indicated. Sleep quality and self-rated health were each measured on a five-point scale (1 = poor, 5 = excellent). ‡ Abuse refers to self-reported exposure of the participant to verbal or physical abuse; the questionnaire did not identify whether the perpetrator was the spouse/partner or another family member. PHQ-2: Two-item Patient Health Questionnaire.

Characteristic	Category	No. (%) or Mean ± SD
Abuse in the previous 12 months ‡	Yes	264 (53.4)
	No	230 (46.6)
Negative life circumstances	Severe family disputes	140 (28.3)
	Severe financial problems	130 (26.3)
	Change of residence	80 (16.2)
	Death of a close person	34 (6.9)
	Difficult health situation	28 (5.7)
	Academic problems	4 (0.8)
	None	78 (15.8)
Little interest or pleasure (PHQ-2)	Not at all	139 (28.1)
	Several days	225 (45.5)
	More than half the days	61 (12.3)
	Nearly every day	69 (14.0)
Feeling down or hopeless (PHQ-2)	Not at all	195 (39.5)
	Several days	169 (34.2)
	More than half the days	86 (17.4)
	Nearly every day	44 (8.9)
Self-rated sleep quality	Mean ± SD (range)	3.3 ± 1.0 (1–5)
Self-rated overall health	Mean ± SD (range)	3.6 ± 1.2 (1–5)

On the RAS, the majority of participants reported high marital satisfaction (363, 73.5%), while 91 (18.4%) had low and 40 (8.1%) had average satisfaction (Figure 1); the mean total RAS score was 24.6 (SD 6.8; range 10-35), and item-level descriptive statistics (Table 4). For physical activity, 367 (74.3%) participants were classified in the low overall IPAQ-SF category, 118 (23.9%) in the moderate category, and 9 (1.8%) in the high category (Figure 2); the distribution of each activity domain and sitting time (Table 5). Only a small number of participants engaged in frequent vigorous activity, and daily sitting time exceeded seven hours in 148 (30.0%) participants.

**Table 4:** Descriptive statistics of the Relationship Assessment Scale (RAS) items and total score among the study participants (N = 494). Values are mean ± SD; each item was rated 1–5. ¶ Reverse-scored before computing the total. § Additional items were collected but not included in the 7-item RAS total score. The total RAS score (range 7–35) was computed from the seven standard items; higher scores indicate greater satisfaction (Cronbach’s alpha = 0.94).

Item	Mean ± SD
Emotional and physical needs met	3.4 ± 1.1
Overall relationship satisfaction	3.6 ± 1.1
Relationship compared with other couples	3.4 ± 1.2
Regret entering the relationship ¶	2.4 ± 1.1
Relationship meets original expectations	3.5 ± 1.0
Love for partner	3.6 ± 1.4
Experiencing relationship problems ¶	2.4 ± 1.1
Satisfaction with closeness and harmony §	3.4 ± 1.1
Partner meets needs in general §	3.5 ± 1.2
Total RAS score (7 items)	24.6 ± 6.8 (range 10–35)

**Figure 1:**
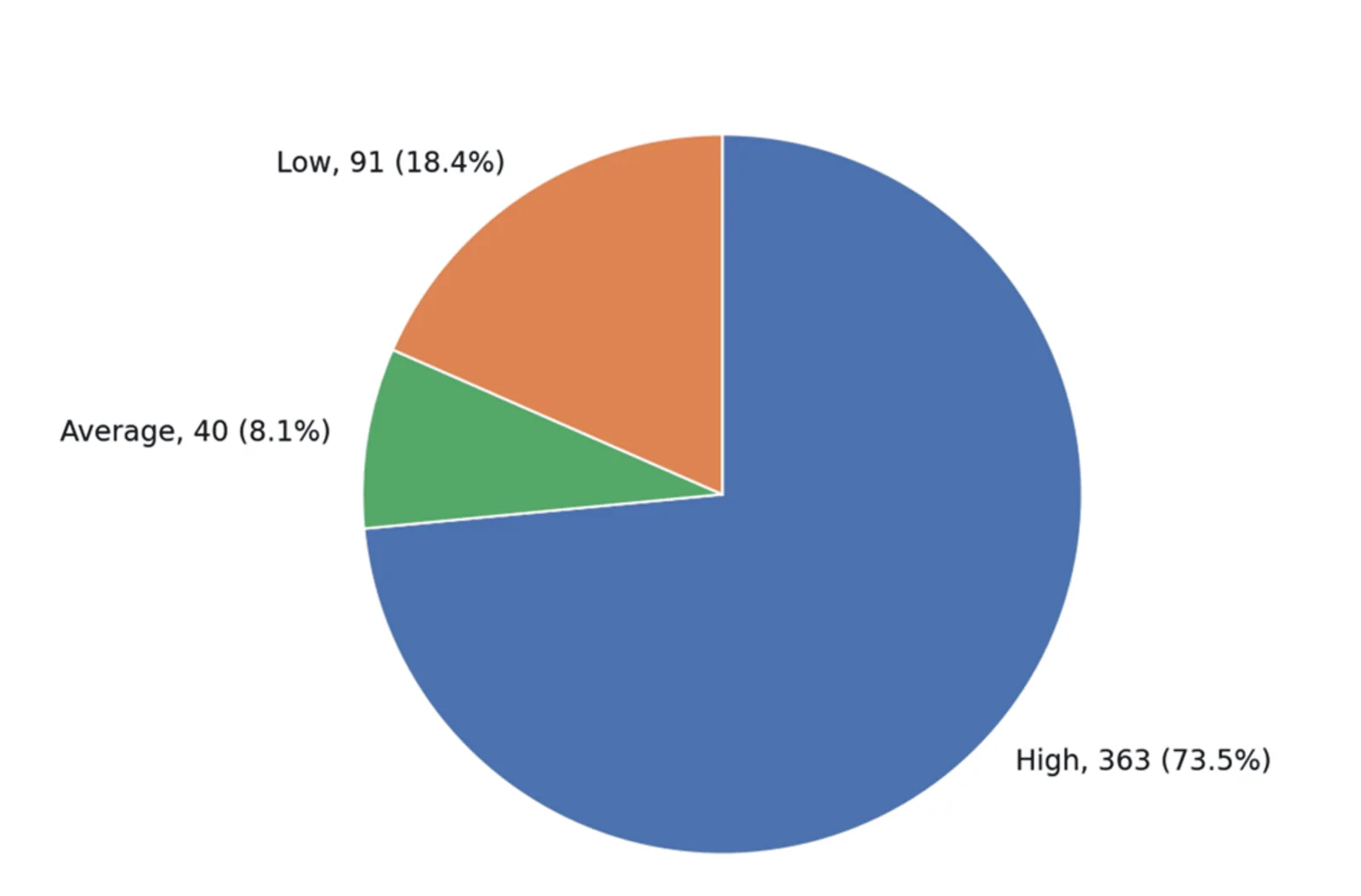
Distribution of participants by marital satisfaction level based on the Relationship Assessment Scale (RAS) (N = 494).

**Figure 2:**
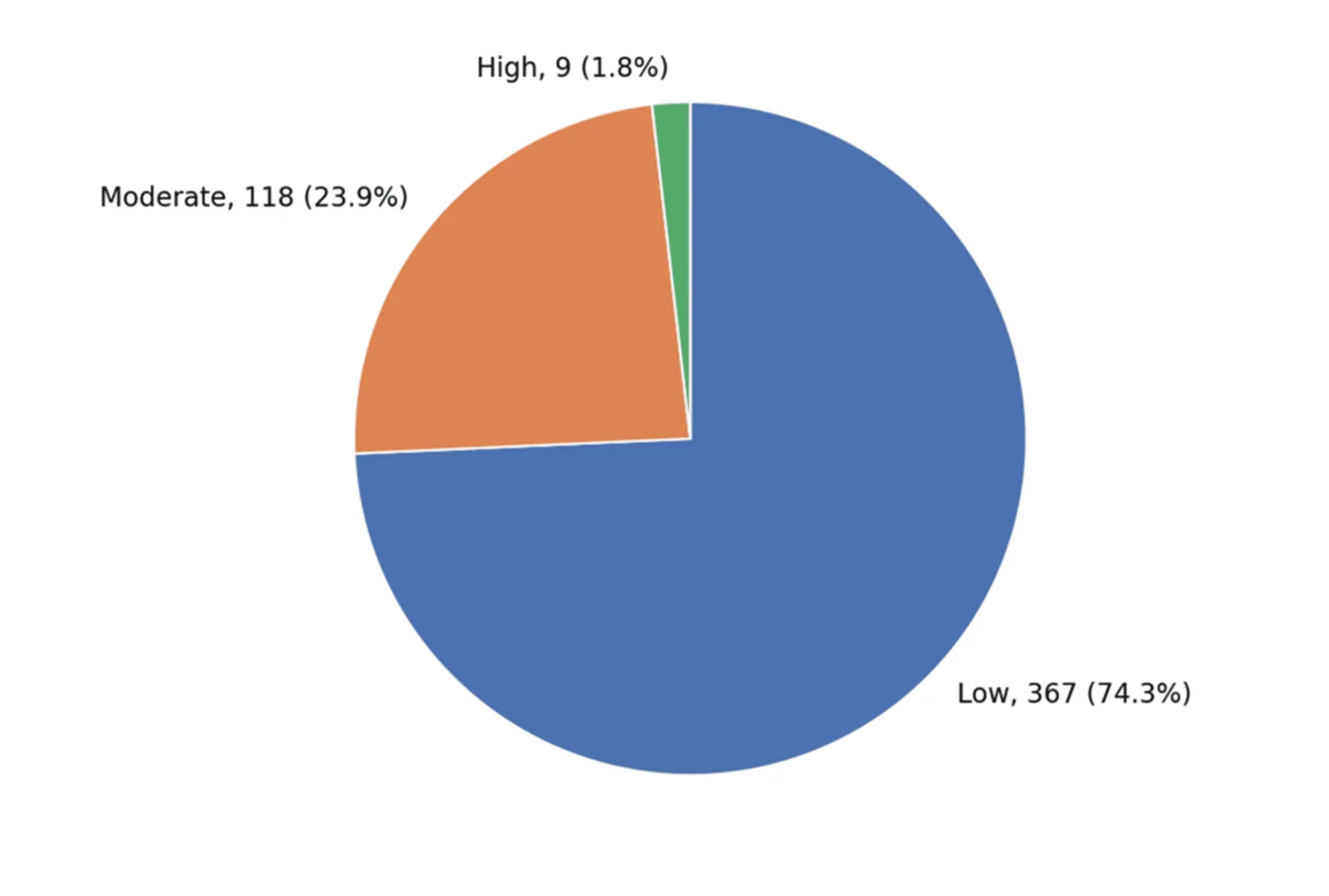
Distribution of the overall IPAQ-SF physical activity category among participants (N = 494). IPAQ-SF: International Physical Activity Questionnaire–Short Form.

**Table 5:** Distribution of physical activity and sedentary behavior among the study participants (N = 494). Values are n (%) unless otherwise indicated. The overall IPAQ-SF physical activity category (low, moderate, high) was derived from the reported weekly frequency of activity, as described in the Methods. IPAQ-SF: International Physical Activity Questionnaire–Short Form.

Measure	Category	No. (%) or Mean ± SD
Vigorous activity (days/week)	None	51 (10.3)
	1–2 days	420 (85.0)
	3–4 days	23 (4.7)
	Mean ± SD	1.2 ± 0.7
Moderate activity (days/week)	None	2 (0.4)
	1–2 days	184 (37.2)
	3–4 days	288 (58.3)
	≥5 days	20 (4.0)
	Mean ± SD	2.8 ± 1.0
Walking (days/week)	None	4 (0.8)
	1–2 days	33 (6.7)
	3–4 days	314 (63.6)
	≥5 days	143 (28.9)
	Mean ± SD	3.9 ± 1.1
Sitting time (hours/day)	<3	4 (0.8)
	3–<5	123 (24.9)
	5–7	212 (42.9)
	>7	148 (30.0)
	Do not know	7 (1.4)
Overall IPAQ-SF physical activity category	Low	367 (74.3)
	Moderate	118 (23.9)
	High	9 (1.8)

In bivariate analysis, marital satisfaction was associated with several sociodemographic and health factors (Table 6): Partner’s age (χ²[4] = 9.97, p = 0.037, V = 0.08), sex (χ²[2] = 19.12, p < 0.001, V = 0.19, with higher satisfaction among men), consanguinity (χ²[2] = 8.34, p = 0.015, V = 0.11), chronic health problems (χ²[2] = 11.73, p = 0.003, V = 0.14), marriage duration (χ²[8] = 45.11, p < 0.001, V = 0.19), and negative life circumstances (χ²[12] = 43.68, p < 0.001, V = 0.18). Participant age (χ²[6] = 12.91, p = 0.070, V = 0.08), educational level, monthly income (p = 0.054), residence, housing, body mass index, smoking, and reported abuse were not significantly associated with marital satisfaction. Sparse-table p-values were obtained from the Fisher-Freeman-Halton exact test. All effect sizes were small to negligible, and the partner’s age association, which was based on sparse cells and had a negligible effect size, should be interpreted cautiously.

**Table 6:** Association between sociodemographic and health-related factors and marital satisfaction among the study participants (N = 494). Values are n (%) by row. The χ² (df) column gives the descriptive Pearson chi-square statistic with degrees of freedom. ^: p-value from the Fisher–Freeman–Halton exact test with Monte Carlo estimation (1,000,000 simulations; seed 2024), used where more than 20% of cells had expected counts <5; other p-values are from the Pearson chi-square test. * p < 0.05 (significant). Effect sizes are bias-corrected Cramér’s V (V < 0.1 negligible, 0.1–0.3 small). SR: Saudi Riyals.

Category	Sub-category	Relationship Satisfaction			χ² (df)	p-value	Cramér’s V
		Low n (%)	Average n (%)	High n (%)			
Age (years)	25–30	4 (13.3)	1 (3.3)	25 (83.3)	12.91 (6)	0.070^	0.08
	31–40	78 (18.4)	31 (7.3)	315 (74.3)			
	41–50	9 (23.7)	8 (21.1)	21 (55.3)			
	51–59	0 (0.0)	0 (0.0)	2 (100.0)			
Partner’s age (years)	25–30	4 (7.0)	7 (12.3)	46 (80.7)	9.97 (4)	0.037*^	0.08
	31–40	87 (20.3)	33 (7.7)	308 (72.0)			
	41–50	0 (0.0)	0 (0.0)	9 (100.0)			
Sex	Male	38 (12.5)	25 (8.2)	242 (79.3)	19.12 (2)	<0.001*	0.19
	Female	53 (28.0)	15 (7.9)	121 (64.0)			
Educational level	Diploma	1 (5.9)	2 (11.8)	14 (82.4)	3.47 (4)	0.575^	0
	University	90 (19.0)	38 (8.0)	345 (72.9)			
	Post-graduate	0 (0.0)	0 (0.0)	4 (100.0)			
Partner’s education	Secondary/below	5 (27.8)	0 (0.0)	13 (72.2)	8.83 (6)	0.122^	0.05
	Diploma	5 (8.2)	8 (13.1)	48 (78.7)			
	University	80 (19.4)	32 (7.8)	300 (72.8)			
	Post-graduate	1 (33.3)	0 (0.0)	2 (66.7)			
Consanguineous marriage	Yes	20 (11.6)	15 (8.7)	138 (79.8)	8.34 (2)	0.015*	0.11
	No	71 (22.1)	25 (7.8)	225 (70.1)			
Monthly income (SR)	3000–6000	0 (0.0)	0 (0.0)	9 (100.0)	15.38 (8)	0.054^	0.09
	6000–10000	7 (10.0)	2 (2.9)	61 (87.1)			
	10000–15000	81 (20.5)	36 (9.1)	279 (70.5)			
	15000–20000	3 (23.1)	2 (15.4)	8 (61.5)			
	>20000	0 (0.0)	0 (0.0)	6 (100.0)			
Chronic health problem	No	13 (10.2)	6 (4.7)	108 (85.0)	11.73 (2)	0.003*	0.14
	Yes	78 (21.3)	34 (9.3)	255 (69.5)			
Marriage duration (years)	1–3	2 (5.7)	1 (2.9)	32 (91.4)	45.11 (8)	<0.001*	0.19
	4–5	10 (12.0)	5 (6.0)	68 (81.9)			
	6–7	19 (11.0)	16 (9.2)	138 (79.8)			
	8–10	51 (29.1)	11 (6.3)	113 (64.6)			
	>10	9 (32.1)	7 (25.0)	12 (42.9)			
Residence area	Urban	71 (18.7)	30 (7.9)	278 (73.4)	0.16 (2)	0.925	0
	Rural	20 (17.4)	10 (8.7)	85 (73.9)			
Housing style	Owner	57 (19.9)	20 (7.0)	209 (73.1)	4.05 (4)	0.526^	0.01
	Rental	34 (16.8)	20 (9.9)	148 (73.3)			
	With family	0 (0.0)	0 (0.0)	6 (100.0)			
Body mass index	Normal	17 (17.3)	5 (5.1)	76 (77.6)	2.91 (4)	0.729^	0
	Overweight	73 (18.8)	35 (9.0)	280 (72.2)			
	Obese	1 (12.5)	0 (0.0)	7 (87.5)			
Abuse (past 12 months)	Yes	41 (15.5)	27 (10.2)	196 (74.2)	5.79 (2)	0.055	0.09
	No	50 (21.7)	13 (5.7)	167 (72.6)			
Negative circumstances	Severe family disputes	25 (17.9)	10 (7.1)	105 (75.0)	43.68 (12)	<0.001*^	0.18
	Change of residence	15 (18.8)	15 (18.8)	50 (62.5)			
	Difficult health situation	10 (35.7)	1 (3.6)	17 (60.7)			
	Severe financial problems	31 (23.8)	11 (8.5)	88 (67.7)			
	Death of a close person	7 (20.6)	3 (8.8)	24 (70.6)			
	Academic problems	0 (0.0)	0 (0.0)	4 (100.0)			
	None	3 (3.8)	0 (0.0)	75 (96.2)			

Marital satisfaction was significantly associated with each physical activity measure (Table 7): vigorous activity (χ²[4] = 53.41, p < 0.001, V = 0.22), moderate activity (χ²[6] = 21.73, p = 0.002, V = 0.13), walking (χ²[6] = 30.71, p < 0.001, V = 0.16), and the overall IPAQ-SF category (χ²[4] = 21.43, p < 0.001, V = 0.13). The direction of these associations indicated that high marital satisfaction was least common among the small groups reporting the most frequent or most vigorous activity; for example, only 2 of 23 participants who reported vigorous activity three to four days per week, and 1 of 9 participants in the high overall category, had high marital satisfaction.

**Table 7:** Association between physical activity and marital satisfaction among the study participants (N = 494). Values are n (%) by column within each marital-satisfaction group. All four measures had sparse cells, so p-values were obtained from the Fisher–Freeman–Halton exact test with Monte Carlo estimation (1,000,000 simulations; seed 2024); the χ² (df) column gives the descriptive Pearson chi-square statistic with degrees of freedom. * p < 0.05 (significant). Effect sizes are bias-corrected Cramér’s V. Crude and adjusted odds ratios for these associations are presented in Table 8. IPAQ-SF: International Physical Activity Questionnaire–Short Form.

Physical activity	Category	Relationship satisfaction			χ² (df)	p-value	Cramér’s V
		Low n (%)	Average n (%)	High n (%)			
Vigorous activity (days/week)	None	10 (11.0)	5 (12.5)	36 (9.9)	53.41 (4)	<0.001*^	0.22
	1–2	67 (73.6)	28 (70.0)	325 (89.5)			
	3–4	14 (15.4)	7 (17.5)	2 (0.6)			
Moderate activity (days/week)	None	1 (1.1)	0 (0.0)	1 (0.3)	21.73 (6)	0.002*^	0.13
	1–2	40 (44.0)	17 (42.5)	127 (35.0)			
	3–4	45 (49.5)	17 (42.5)	226 (62.3)			
	≥5	5 (5.5)	6 (15.0)	9 (2.5)			
Walking (days/week)	None	4 (4.4)	0 (0.0)	0 (0.0)	30.71 (6)	<0.001*^	0.16
	1–2	12 (13.2)	1 (2.5)	20 (5.5)			
	3–4	44 (48.4)	26 (65.0)	244 (67.2)			
	≥5	31 (34.1)	13 (32.5)	99 (27.3)			
Overall IPAQ-SF category	Low	67 (73.6)	24 (60.0)	276 (76.0)	21.43 (4)	<0.001*^	0.13
	Moderate	19 (20.9)	13 (32.5)	86 (23.7)			
	High	5 (5.5)	3 (7.5)	1 (0.3)			

Crude and adjusted associations from logistic regression (Table 8). In the primary model, compared with the low physical activity category, the moderate category was not associated with high marital satisfaction (aOR 0.96, 95% CI 0.58-1.57), whereas the high category showed markedly lower odds of high satisfaction (aOR 0.07, 95% CI 0.01-0.45); the latter estimate is imprecise and should be interpreted with caution because only nine participants were in the high category. Modeled as an ordinal (per-category) term, a higher overall IPAQ-SF category was associated with modestly lower odds of high marital satisfaction (crude OR 0.61, 95% CI 0.41-0.90; aOR 0.66, 95% CI 0.44-1.00, p = 0.048). In the secondary domain models, more frequent vigorous activity was inversely and significantly associated with high marital satisfaction (aOR per level 0.44, 95% CI 0.24-0.79, p = 0.006), whereas moderate activity (aOR 1.12, 95% CI 0.77-1.62) and walking (aOR 1.24, 95% CI 0.87-1.76) were not independently associated.

**Table 8:** Crude and adjusted associations between physical activity and high marital satisfaction (high versus low/average) among the study participants (N = 494). The outcome was high marital satisfaction versus low/average satisfaction. Adjusted models included age, sex, monthly income, and chronic health problems, selected a priori. Firth’s penalized likelihood was used for the categorical overall-category model because of sparse data. The activity domains were entered in separate secondary models. ¶ Only nine participants were in the high category; this estimate is imprecise and should be interpreted with caution. † Modeled as an ordinal (per-category) term to provide a stable estimate. OR: odds ratio; CI: confidence interval; IPAQ-SF: International Physical Activity Questionnaire–Short Form.

Exposure	Comparison	Crude OR (95% CI)	Adjusted OR (95% CI)	p (adjusted)
Overall IPAQ-SF category	Moderate vs low	0.88 (0.55–1.41)	0.96 (0.58–1.57)	0.86
	High vs low ¶	0.06 (0.01–0.37)	0.07 (0.01–0.45)	0.006
Overall IPAQ-SF category	Per category increase †	0.61 (0.41–0.90)	0.66 (0.44–1.00)	0.048
Vigorous activity	Per level increase †	0.36 (0.20–0.64)	0.44 (0.24–0.79)	0.006
Moderate activity	Per level increase †	1.12 (0.78–1.60)	1.12 (0.77–1.62)	0.56
Walking	Per level increase †	1.13 (0.81–1.58)	1.24 (0.87–1.76)	0.24

Physical activity did not differ significantly by sex (χ²[2] = 1.33, p = 0.52), age group (χ²[6] = 10.45, p = 0.058), or body mass index category (χ²[4] = 0.77, p = 0.95) (Table 9). The sex × physical activity interaction was not significant (p = 0.76), so results are presented for the combined sample adjusted for sex rather than separately by sex. Non-smoking was more common among women (89.9%) than men (79.0%) (χ²[2] = 10.07, p = 0.007), and smoking itself was not associated with marital satisfaction (p = 0.99). Reported exposure to abuse in the previous 12 months was not associated with marital satisfaction: high satisfaction was reported by 196 of 264 (74.2%) participants who reported abuse and 167 of 230 (72.6%) who did not (χ²[2] = 5.79, p = 0.055, V = 0.09). In the sensitivity analysis excluding the two participants who reported more than one wife (N = 492), the adjusted estimates were essentially unchanged (e.g., high versus low category aOR 0.07, 95% CI 0.01-0.46), indicating that the results were robust to these participants. When marital satisfaction was modeled as an ordinal outcome, the direction and magnitude were consistent with the primary analysis (OR 0.71, 95% CI 0.48-1.05, p = 0.09), although the proportional-odds assumption was only partially met, so the binary model was retained as the primary analysis.

**Table 9:** Distribution of the overall IPAQ-SF physical activity category by sex, age group, and body mass index (N = 494). Values are n (%) by row. Physical activity did not differ significantly by sex, age group, or body mass index. The age-group and body mass index comparisons used the Fisher–Freeman–Halton exact test (Monte Carlo, 1,000,000 simulations, seed 2024; marked ^) because more than 20% of cells had expected counts <5; the sex comparison used the Pearson chi-square test. For reference, non-smoking was more common among women (170/189, 89.9%) than men (241/305, 79.0%) (χ²(2) = 10.07, p = 0.007), and smoking was not associated with marital satisfaction (p = 0.99). PA: Physical activity; IPAQ-SF: International Physical Activity Questionnaire–Short Form.

Subgroup	Category	Low PA n (%)	Moderate PA n (%)	High PA n (%)	Test
Sex	Male	232 (76.1)	68 (22.3)	5 (1.6)	χ²(2) = 1.33, p = 0.52
	Female	135 (71.4)	50 (26.5)	4 (2.1)	
Age group (years)	25–30	18 (60.0)	12 (40.0)	0 (0.0)	χ²(6) = 10.45, p = 0.058^
	31–40	324 (76.4)	93 (21.9)	7 (1.7)	
	41–50	24 (63.2)	12 (31.6)	2 (5.3)	
	51–59	1 (50.0)	1 (50.0)	0 (0.0)	
Body mass index	Normal weight	72 (73.5)	25 (25.5)	1 (1.0)	χ²(4) = 0.77, p = 0.950^
	Overweight	289 (74.5)	91 (23.5)	8 (2.1)	
	Obese	6 (75.0)	2 (25.0)	0 (0.0)	

## Discussion

In this cross-sectional study of married Saudi adults attending primary health care centers, most participants reported high marital satisfaction, while physical activity was generally low. Marital satisfaction was statistically associated with each physical activity measure, but the effect sizes were small, and the direction was the opposite of a simple “more activity, more satisfaction” pattern. After adjustment, a higher overall IPAQ-SF category and, in particular, more frequent vigorous activity were associated with lower odds of high marital satisfaction, whereas moderate activity and walking showed no independent association. These associations were driven largely by the small groups of participants who reported the most frequent or most vigorous activity, among whom high satisfaction was uncommon.

This pattern differs from some previous reports that have described positive links between physical activity and relationship or psychological well-being [11,17-20]. Several explanations are possible. First, the number of participants engaging in high or vigorous activity was very small, so the corresponding estimates are imprecise and may reflect the particular characteristics of a few individuals rather than a stable relationship. Second, because the design is cross-sectional, the association may reflect reverse or bidirectional processes; for example, individuals experiencing lower relationship satisfaction might invest more time in vigorous exercise, rather than exercise reducing satisfaction. Third, most participants were classified as having low physical activity, which limited the contrast available to detect a graded association. Taken together, the findings indicate that, in this population, physical activity was at most weakly and inconsistently associated with marital satisfaction.

Consistent with previous research, marital satisfaction in this sample was associated with sex, marriage duration, and chronic health problems, in line with determinants reported in Middle Eastern and Saudi populations [21-23], with men reporting higher satisfaction than women [24,25] and satisfaction being lower in longer-duration marriages [26-28]. The observed decline in satisfaction with longer marriage duration is in keeping with the U-shaped or declining trajectories described in longitudinal work [28,29]. Physical activity itself did not differ significantly by sex, age group, or body mass index, and the sex × physical activity interaction was not significant, so a single combined model adjusted for sex was appropriate rather than sex-specific models.

Reported exposure to abuse in the previous 12 months was common but was not associated with marital satisfaction. This measure captured self-reported verbal or physical abuse and did not identify whether the source was the spouse/partner or another family member; it should therefore not be interpreted as spousal abuse. The absence of an association, together with this measurement limitation, means that the coexistence of frequently reported abuse and high marital satisfaction should be interpreted cautiously and does not by itself indicate a paradox.

From a public-health perspective, the most consistent observation was the low level of physical activity and the high proportion of participants with prolonged daily sitting time, which is relevant given the well-established health benefits of regular activity [8,30]. This supports continued efforts to promote physical activity in this population, independent of its uncertain relationship with marital satisfaction.

The strengths of this study include a relatively large sample recruited through multistage stratified random sampling, the use of standardized instruments with good internal consistency for the RAS, and the reporting of effect sizes and sensitivity analyses in addition to statistical tests. Several limitations should be considered. The cross-sectional design does not allow the temporal direction of the association to be determined; it is not possible to establish whether physical activity influences marital satisfaction, whether marital satisfaction influences physical activity, or whether both are driven by unmeasured factors, and reverse causation and bidirectionality are possible. The reliance on self-reported measures introduces potential recall and social-desirability bias, and because duration in minutes was largely missing, physical activity was classified from reported frequency rather than the standard MET-based scoring, which limits comparability with studies using full IPAQ-SF scoring. The small numbers of participants in the high and vigorous activity categories produced imprecise estimates, as reflected in the wide confidence intervals. Participants were recruited from primary health care attendees in Taif, who may differ from the general married adult population, limiting generalizability; the number of eligible visitors who were approached but declined was not recorded, so a participation rate could not be calculated. Eligibility required marriage for at least two years; six respondents who reported a marriage duration of less than one year were excluded, but because marriage duration was recorded only in categories, the retained “1-3 years” category could not separate participants married for one to less than two years from those married for at least two years, so a small degree of residual eligibility misclassification cannot be excluded. Finally, although key confounders were considered, residual confounding cannot be excluded.

## Conclusions

In this cross-sectional study of married Saudi adults in Taif, most participants reported high marital satisfaction, whereas physical activity levels were generally low. Physical activity was only modestly associated with marital satisfaction: more frequent vigorous activity, and the very small high overall activity category (only nine participants), were inversely associated with high satisfaction, while moderate activity and walking showed no independent association. Because the study was cross-sectional and several estimates were imprecise, these findings should be interpreted as associations rather than causal effects, and the direction of any relationship cannot be established. The low overall level of physical activity in this population is itself a relevant target for health promotion. Longitudinal studies are needed to clarify the direction and mechanisms of the relationship between physical activity and marital satisfaction.
